# Early impact of the Inflation Reduction Act on small molecule vs biologic post-approval oncology trials

**DOI:** 10.1093/haschl/qxaf152

**Published:** 2025-08-28

**Authors:** Hanke Zheng, Julie A Patterson, Jonathan D Campbell

**Affiliations:** Research Department, National Pharmaceutical Council, Washington, DC 20006, United States; Research Department, National Pharmaceutical Council, Washington, DC 20006, United States; Research Department, National Pharmaceutical Council, Washington, DC 20006, United States

**Keywords:** Inflation Reduction Act, Drug Price Negotiation Program, post-approval clinical development, oncology, small molecule

## Abstract

**Introduction:**

Under the Inflation Reduction Act (IRA), small molecule drugs are subject to a shorter timeline toward eligibility for selection to the Drug Price Negotiation Program (DPNP) than biologics (7 vs 11 years post-approval), raising concerns about incentives for post-approval clinical development.

**Methods:**

Using Citeline's Trialtrove database (7/2014-8/2024), this longitudinal study explored the impact of IRA's passage on industry-sponsored, post-approval phase I-III clinical trials in small molecule vs biologic oncology drugs, excluding vaccine-related trials. We used a difference-in-difference design to explore the impact of the IRA's differential DPNP timeline on small molecule trials in oncology by comparing changes in the number of newly initiated post-approval trials in small molecule drugs after the IRA (first difference) with changes in biological trials (second difference).

**Results:**

The monthly average of small molecule and biologic trials dropped by 45.3% (*P* < .01) and 32.5% (*P* < .01) post-IRA, respectively. Compared with biologics, small molecules were associated with an additional decrease of 4.5 trials/month (−4.5, 95% CI, −7.1 to −1.9; *P* < .01) after the IRA's passage.

**Conclusion:**

This finding supports hypotheses that the IRA's differential timelines toward DPNP eligibility for the 2 molecule types may disproportionately disincentivize post-approval research in small molecule drugs.

Key points

- The Inflation Reduction Act (IRA) was associated with a decline in industry-funded post-approval oncology trials.
- Following the IRA, small molecule oncology drugs experienced a disproportionately greater reduction in post-approval clinical trial initiations, compared with biologic drugs.
- These findings support concerns around the disincentivizing effect of the IRA and its shorter price negotiation eligibility timeline for small molecules on post-approval research in oncology.

## Introduction

The Inflation Reduction Act (IRA) of 2022 changed the landscape of drug pricing in the United States by introducing a new timeline toward anticipated price erosion for drugs selected for the IRA's Drug Price Negotiation Program (DPNP).^1^ The DPNP grants the Centers for Medicare and Medicaid Services authority to establish “maximum fair prices” for selected drugs, which cannot exceed statutorily defined maximum ceiling prices based on a percentage of nonfederal average manufacturer prices for Part D drugs or average sales price for Part B drugs. The timeline from a drug's initial Food and Drug Administration (FDA) approval to its eligibility for DPNP selection differs between small molecule (ie, 7 years) and biologic (ie, 11 years) drugs.^1^

The potential impact of the IRA on research and development (R&D) has been widely debated in literature, often in the context of post-approval development and the law's shorter DPNP eligibility timeline for small molecules.^2-4^ Post-approval clinical trials and FDA approvals of subsequent indications frequently occur around or after the point when drugs become eligible for DPNP selection, particularly among small molecules. Among recently approved small molecule cardiovascular and oncology drugs, a reported 25% and 46% of subsequent indications, respectively, were approved 7 or more years after initial FDA approval.^5^

Early evidence of the IRA's impact on R&D across therapeutic areas has analyzed the law's impact on small molecule development, suggesting larger declines in both early-stage venture capital investments and post-approval clinical trials in small molecules than biologics following the law's passage.^4,6,7^ These potential impacts are particularly relevant in oncology, given recent increases in targeted small molecule therapies, the frequency of subsequent indications, and high Medicare utilization among cancer drugs.^3,5,8,9^ The study aimed to add to the limited existing research exploring the impact of the IRA's shorter timelines toward DPNP eligibility for small molecules, with a focus on oncology. Specifically, we aimed to estimate the impact of the IRA and its differential DPNP eligibility timelines on the initiation of post-approval clinical trials among oncology small molecule drugs, using biologic trials as the comparison group.

## Methods

Using Citeline's Trialtrove database, we identified industry-funded phase I-III trials initiated between 7/2014 and 8/2024 for approved oncology drugs, excluding trials for cancer vaccines. Trialtrove provides a comprehensive and rigorously curated source of registered drug trial data, offering detailed insights into trial design, endpoints, outcomes, and other key trial characteristics. Data were extracted in 4/2025, when the most recently curated data available were from 8/2024. The study sample included trials with primary tested drug(s) that were exclusively either small molecule or biologic drugs. Trials for small molecules vs biologics were determined by Citeline classification and adjudication by 2 PharmD reviewers.

In this retrospective data analysis, we first described the monthly average number of trials before and after the IRA's passage in the sample of all oncology post-approval trials, as well as the subgroups of trials for small molecule drugs and biologics. Pre- and post-IRA periods were defined as 7/2014 to 7/2022 and 8/2022 to 8/2024, respectively. Sensitivity analyses explored potential confounding effects of COVID-19. First, a shorter time horizon was studied to isolate the study period to the post-COVID-19 landscape, comparing the year prior to the IRA's passage (8/2021 to 7/2022) to the most recent extracted year (9/2023 to 8/2024). Second, clinical trial initiation was described during 3 time periods: pre-COVID-19 (7/2014 to 2/2020), COVID-19 to IRA (3/2020 to 7/2022), and post-IRA (8/2022 to 8/2024). Wilcoxon rank-sum tests were then used to test for pre/post differences overall and within the 2 subgroups.

Next, we used a quasi-experimental difference-in-difference (DiD) design to estimate the impact of the shorter DPNP eligibility timeline on initiation of post-approval clinical trials among oncology small molecule drugs, using biologic trials to model the counterfactual trajectory—that is, the expected trend in small molecule trials had they not been subject to a shorter DPNP timeline. The intervention was the passage of the IRA's shorter timeline toward DPNP eligibility for small molecule drugs than biologics. The study outcome was the monthly number of industry-sponsored, post-approval trial initiations. The DiD approach aimed to isolate the effect of the shorter DPNP timeline on post-approval development in small molecule drugs by comparing differences in the newly initiated trials over time between small molecule and biologic drugs. The underlying assumption was that post-approval clinical development would have followed a similar trajectory in both small molecules and biologics in the absence of IRA's differential DPNP eligibility timeline. Thus, the DiD framework mitigates the observed and unobserved confounding factors that influenced both small molecule and biologic trials around the time of the IRA's passage, for example, the potential lagging effect of the COVID-19 pandemic and the evolving financial environment alongside increases in the Federal Reserve interest rates between 3/2022 and 7/2023.

For the DiD estimate, we constructed a linear regression with an indicator for small molecule trials, a post-IRA indicator, an interaction between the small molecule trial and the post-IRA indicators (the DiD estimator), and the time-fixed effect to account for secular trends unrelated to the IRA and the differential DPNP timeline. Heteroskedasticity-robust standard errors were used. We conducted visual inspections and statistical analyses to test for the parallel trends assumption in the pre-IRA period. Specifically, we first descriptively examined the outcome of interest over time before the IRA's passage. Second, a linear regression was conducted to test for time-varying trends between small molecule and biologic trials before the IRA; 10/2014 to 7/2022 was determined as the pre-IRA period for the main DiD model based on parallel trends assumption tests. Third, we used a shorter post-COVID-19 period (1/2020 to 7/2022) in the DiD model to test the result robustness to changes in the chosen pre-IRA period.

Data analyses were conducted using *Stata SE, v18*. This study was exempt from Institutional Review Board review as it did not involve patient-level data or human subjects research.

## Results

### Study sample and descriptive findings

**Figure 1.**
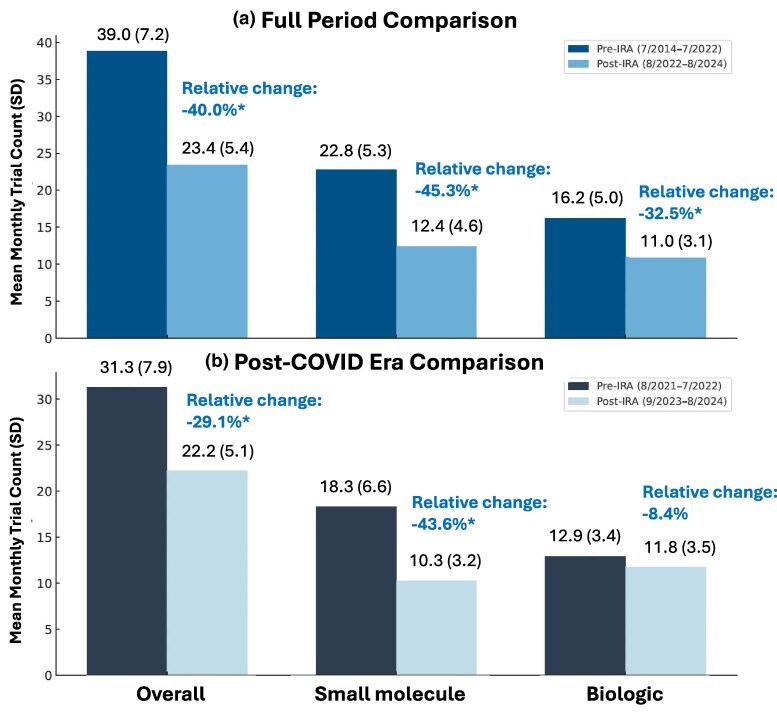
Monthly number of trials before and after the passage of the Inflation Reduction Act, overall and by molecule type: (a) full period comparison, and (b) post-COVID era comparison. SD, standard deviation; IRA, Inflation Reduction Act. *P < .01.

Applying the selection criteria, we identified 4367 industry-sponsored post-approval phase I-III trials in oncology from 7/2014 to 8/2024, with 57.7% (*n* = 2519) testing small molecules, 42.3% (*n* = 1848) testing biologic drugs. Across the full period (7/2014 to 7/2022 vs 8/2022 to 8/2024), the monthly average of all post-approval oncology trials decreased by 40.0% (*P* < .01) after the IRA's passage. Small molecule and biologic trials dropped by 45.3% (*P* < .01) and 32.5% (*P* < .01) post-IRA, respectively (Figure 1). In the post-COVID-19 era sensitivity analysis (8/2021 to 7/2022 vs 9/2023 to 8/2024), post-approval oncology trials dropped by 29.1% (*P* < .01). The monthly average of post-approval clinical trials in small molecule drugs decreased by 43.6% (*P* < .01), with a non-statistically significant reduction of 8.4% in biologic trials (*P* = .45). In the 3-period sensitivity analysis, trial initiation numerically declined from COVID-19 to IRA for both small molecules (−10.6%; Supplementary material) and biologics (−7.8%), with further and numerically larger declines post-IRA (small molecules: −41.0%; biologics: −28.1%).

### DiD estimates and sensitivity analyses

**Figure 2.**
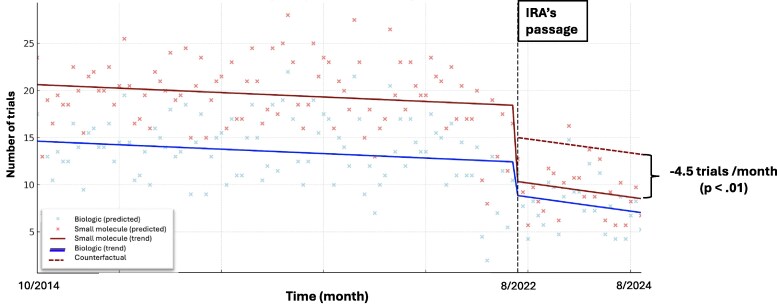
Difference-in-difference model estimating the impact of the Inflation Reduction Act's differential drug price negotiation program timeline on post-approval trials for oncology small molecule drugs. IRA, Inflation Reduction Act; DPNP, Drug Price Negotiation Program. The intervention was the passage of the IRA, which included a differential timeline toward DPNP eligibility for small molecule and biologic drugs, in 8/2022; the trend of biologic trials was used to model the counterfactual trajectory—that is, the expected trend in small molecule trials had they not been subject to a shorter DPNP timeline; the parallel trends assumption was tested to select the pre-period (10/2014-7/2022).

According to the DiD estimates (Figure 2), the IRA's shorter DPNP eligibility timeline was associated with an additional reduction of 4.5 trials/month for small molecule oncology drugs (−4.5, 95% CI, −7.1 to −1.9; *P* < .01), compared with the counterfactual modeled by the biologics' trend. The parallel trend assumption was not violated by visual inspection and statistical tests, as the regression in the pre-IRA phase (10/2014 to 7/2022) indicated that the interaction between the small molecule trial indicator and the time variable was not statistically significant. In the pre-IRA period, the trend of post-approval clinical trials was similarly stable for both the small molecules (−0.04 trials/month) and biologics (−0.003 trials/month). Using the shorter pre-IRA period (ie, post-COVID-19) in the DiD model, the sensitivity analysis suggested an additional reduction of 4.4 (−4.4, 95% CI, −7.5 to −1.4; *P* < .01) trials/month for small molecules associated with the IRA's differential DPNP eligibility timeline.

## Discussion

Our study presents evidence of early signals of the impact of the IRA on post-approval clinical development in previously approved oncology drugs. We observed an overall decline in industry-funded post-approval phase I-III oncology trials post-IRA as well as a greater reduction in small molecule trials than biologic trials, likely reflecting the differential timeline toward DPNP eligibility for the 2 molecule types. These findings inform discussions about potential unintended consequences of the IRA on post-approval development in oncology, particularly in small molecule drugs.

Using a large and representative sample of clinical trials, our analysis suggested a decline in industry-funded post-approval oncology trials associated with the IRA. Past studies examining timelines of clinical development in the context of the IRA have raised questions surrounding the potential for the law to disincentivize post-approval research.^3,5^ Incentives around post-approval research are pertinent considerations in oncology, where two-thirds (68%) of industry-funded oncology trials take place after a drug's initial approval and as many as 75% of cancer drugs are approved for multiple indications.^3,10-12^ Our study builds upon early evidence of the IRA's potential impact on post-approval development across therapeutic areas by exploring changes in post-approval clinical trial initiation after the IRA specifically in oncology drugs.^7^

Our findings indicate that the IRA's passage was associated with larger reductions in post-approval trials for small molecule than for biologic oncology drugs. Previous research has highlighted the frequency with which post-approval clinical trials and subsequent indication approvals occur around or after DPNP eligibility, particularly for small molecules, raising concerns created by the IRA's differential timelines.^3,5,10-13^ This study used a quasi-experimental, DiD approach to isolate the effect of the shorter DPNP timeline for small molecule drugs. The model's finding of an additional reduction in trials following the IRA's passage for small molecule oncology drugs supports concerns about the disincentivizing effect of the shorter eligibility timeline. This evidence adds to recent findings of a disproportionate impact of the IRA on small molecules at early stages of investment in therapeutics with high exposure to Medicare-aged populations.^4^ Amid concerns about the potential differential effect of the IRA on small molecule drug development, 2 pieces of legislation have been proposed to equalize DPNP eligibility timelines for all small molecules alongside those with genetically targeted technology.^14,15^

This study is not without limitations. Our study design does not adjust for all potential confounding factors; while study results support a strong association consistent with IRA impacts, future research is needed to further assess causality. While we found no evidence violating the parallel trends assumption, there may be time-varying unobserved factors associated with the observed association between the shorter DPNP's timeline and reduction in post-approval clinical development for small molecule oncology drugs. Furthermore, while we modeled the intervention at the IRA's passage, its influence on investment decisions may have begun earlier, potentially as early as 11/2021 when it first entered formal debate.^1,16^ Moreover, although we excluded phase IV trials, some post-marketing requirements and post-marketing commitments may still be present in our sample, as Citeline's Trialtrove data do not differentiate them from manufacturer-sponsored post-approval trials unrelated to requirements or commitments from past FDA approval(s). As a result, this could lead to an underestimation of the IRA's impact on post-approval clinical development in oncology drugs. Finally, our study could not capture the long-term trends in post-approval clinical development after the IRA's passage and its broader implication on clinical outcomes, Medicare spending, development in specific therapeutic areas, and patient access. Future research is needed to monitor the long-term effects of the law on innovation, affordability, and patient access.

## Conclusion

This study provides early evidence of an overall decline in industry-funded post-approval clinical trials in oncology drugs following the approval of the IRA. Using a quasi-experimental study design to isolate the effect of the shorter DPNP timeline for small molecule drugs, this study suggests a greater post-IRA reduction in small molecule trials than biologic trials. The study findings support a hypothesis that the IRA's differential timelines toward DPNP eligibility for the 2 molecule types disproportionately disincentivizes post-approval research in small molecule drugs. The findings inform further discussions on the potential unintended consequences of the IRA and proposed legislative changes to the policy.

## Supplementary Material

qxaf152_Supplementary_Data
