# Supplementary material for: Early impact of the Inflation Reduction Act on small molecule vs biologic post-approval oncology trials
Source: Health Aff Sch. 2025 Aug 28;3(8):qxaf152. doi: 10.1093/haschl/qxaf152 (PMC12392883; doi:10.1093/haschl/qxaf152)
Supplement: qxaf152_Supplementary_Data [file qxaf152_supplementary_data.zip › Appendix.docx]

**Appendix**

**sFigure 1. Observed Monthly Post-Approval Trial Initiations in Small Molecule and Biologic Oncology Drugs.**


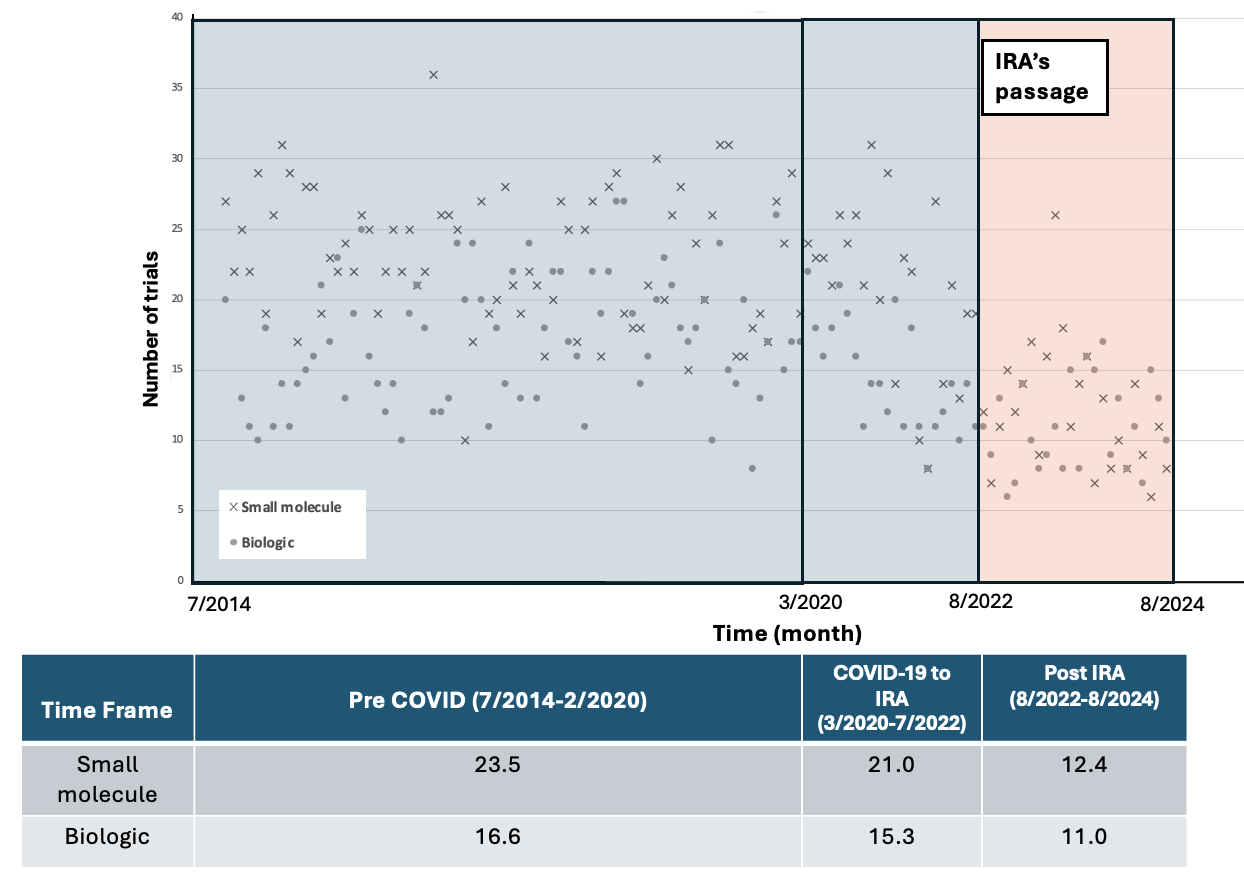


*Notes: IRA, inflation reduction act. Given the exact timing of when COVID-19 began to systematically affect trial initiation and how long that effect persisted is uncertain, we assumed the disruption began in March 2020, consistent with the timing of the World Health Organization’s official designation of COVID-19 as a pandemic, the nationwide emergency declaration in the United States, and state-level shutdowns to prevent the spread of COVID-19.*
